# Microwave absorption properties of Ni/(C, silicides) nanocapsules

**DOI:** 10.1186/1556-276X-7-238

**Published:** 2012-05-01

**Authors:** Jingjing Jiang, Han Wang, Huaihong Guo, Teng Yang, Wen-Shu Tang, Da Li, Song Ma, Dianyu Geng, Wei Liu, Zhidong Zhang

**Affiliations:** 1 Shenyang National Laboratory for Materials Science, Institute of Metal Research, and International Centre for Materials Physics, Chinese Academy of Sciences, 72 Wenhua Road, Shenyang, 110016, People’s Republic of China

**Keywords:** Microwave absorption properties, Magnetic nanocapsules, Complex permittivity, Electric dipole model

## Abstract

The microwave absorption properties of Ni/(C, silicides) nanocapsules prepared by an arc discharge method have been studied. The composition and the microstructure of the Ni/(C, silicides) nanocapsules were determined by means of X-ray diffraction, X-ray photoelectric spectroscopy, and transmission electron microscope observations. Silicides, in the forms of SiO_x_ and SiC, mainly exist in the shells of the nanocapsules and result in a large amount of defects at the ‘core/shell’ interfaces as well as in the shells. The complex permittivity and microwave absorption properties of the Ni/(C, silicides) nanocapsules are improved by the doped silicides. Compared with those of Ni/C nanocapsules, the positions of maximum absorption peaks of the Ni/(C, silicides) nanocapsules exhibit large red shifts. An electric dipole model is proposed to explain this red shift phenomenon.

## Background

Magnetic nanocapsules have attracted increasing attention in the area of electromagnetic wave absorption in the last decades because of their peculiar structural characteristics [1-4]. The magnetic nanocapsules, such as Fe/ZnO [1] and FeCo/C [2] nanocapsules, consist of different core and shell materials, and they show outstanding electromagnetic wave absorption performance in the gigahertz range. Zhang et al. [3,4] have prepared Ni/C nanocapsules using methane as the carbon source and observed excellent electromagnetic properties, and they also studied the effect of inter-particle distance on the electromagnetic wave attenuation mechanism. Recently, Wang et al. (unpublished work) have reported that changing the thickness of the graphite shell of Ni/C nanocapsules can modulate the effective permittivity.

Both carbon and silicon dioxide are widely used as shell materials, in which the former has a layered structure and the latter is a good insulator [5]. SiC with a high dielectric constant is an interesting wave absorption material [6]. As we know, SiO_2_ can react with carbon at high temperatures to produce semiconducting SiC [7]. We expect to improve the electromagnetic wave absorption properties of nanocapsules by changing the shell constituents of nanocapsules. In this paper, the electromagnetic wave absorption properties of Ni/(C, silicides) nanocapsules and Ni/C nanocapsules are studied in the frequency range of 2 to 18 GHz. Although the tendencies of the frequency dependence of the complex permeability of the Ni/(C, silicides) and Ni/C nanocapsules in the frequency range of 2 to 18 GHz are similar, compared with the electromagnetic absorption spectra of the Ni/C nanocapsules, the absorption peak of the Ni/(C, silicides) nanocapsules shows a large red shift of 0.7 to 1.9 GHz at different thicknesses of absorption layer. An electric dipole model is proposed to explain this red shift phenomenon.

## Methods

Ni/(C, silicides) nanocapsules were fabricated by the arc discharge method as described in [8]. A mixture of nickel and silicon dioxide powders with a mole ratio of Ni/SiO_2_ = 96:4 was compacted into a target and used as an anode. A graphite needle served as a cathode. A mixture gas of high-purity Ar (2.0 × 10^4^ Pa) and H_2_ (4.0 × 10^3^ Pa) was introduced into the evacuated chamber (5.0 × 10^−3^ Pa) serving as the source of plasma. Twenty milliliters of 99.7% C_2_H_5_OH was introduced into the chamber as the carbon source. During the experimental process, the current was maintained at 80 A, and the voltage, at 18 V. The products were collected from the chamber after passivation for 12 h in Ar atmosphere. In order to study the effects of inclusions of silicides on the absorption properties of Ni/(C, silicides) nanocapsules, Ni/C nanocapsules were also prepared using a pure Ni target as an anode under the same conditions.

Phase analysis was performed by means of X-ray diffraction (XRD). X-ray photoelectron spectroscopy (XPS) was used to determine the elements’ chemical states and the valance band spectrum. The morphology and microstructure of the samples were characterized using a transmission electron microscope (TEM; JEOL-2100, JEOL Ltd., Akishima, Tokyo, Japan) with an emission voltage of 200 kV. The sample preparation for complex permittivity and permeability measurements is described in detail elsewhere [1,9]. The mass fraction of the nanocapsules in paraffin was set at 50 wt.%. The coaxial method was used to determine the EM parameters of the toroidal samples (see details for the preparation of toroidal samples in [2]) in the frequency range of 2 to 18 GHz using an Agilent 8722ES vector network analyzer (VNA; Santa Clara, CA, USA) with a transverse electromagnetic mode. The complex permittivity and complex permeability were derived from the *S*-parameters tested by the calibrated VNA, using a simulation program for the Reflection/Transmission Mu and Epsilon (Nicholson-Ross-Weir model) [10]. According to the transmission line model [11], the reflection losses (RLs) of the Ni/(C, silicides) and Ni/C nanocapsules were calculated from the complex permittivity and complex permeability measured on the nanocapsules dispersed in paraffin.

## Results and discussion

**Figure 1:**
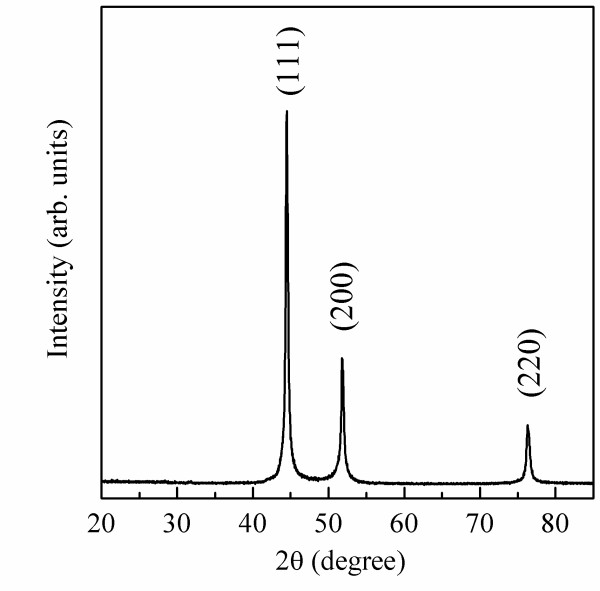
XRD pattern of Ni/(C, silicides) nanocapsules.

The XRD patterns of Ni/(C, silicides) nanocapsules (Figure 1) show Ni reflections, indicating that the main parts of the cores are Ni. No clear reflections of carbon, carbides, and silicides are observed due to their small amount or the amorphous state of the shell structure.

**Figure 2:**
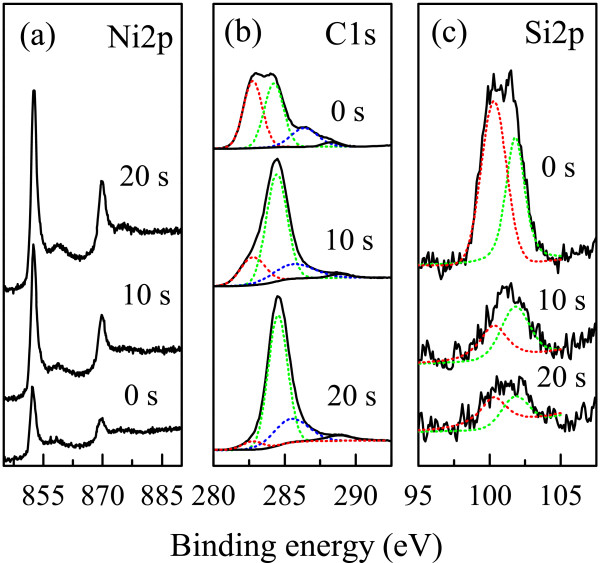
**XPS of (a) Ni2*p*, (b) C1*s*, and (c) Si2*p*in Ni/(C, silicides) nanocapsules.**

In order to determine the surface compositions of the Ni/(C, silicides) nanocapsules, XPS spectra were measured after etching times of 0, 10, and 20 s. The XPS spectra of the Ni/(C, silicides) nanocapsules are shown in Figure 2. The binding energies of 852.3 and 870 eV in Figure 2a correspond to the Ni2*p*_3/2_ and Ni2*p*_1/2_ electron states in metallic Ni cores, while the weak peak at 856.1 eV indicates the satellite peak of Ni2*p*_3/2_. Figure 2b,c shows the binding energy curves of C1*s* and Si2*p*, respectively. The binding energy of 282.2 eV in Figure 2b reveals the C1*s* electron of SiC, and the one at 284.6 eV corresponds to the existence of graphite. It is noted that the intensity of the peak at 282.2 eV weakens harshly after Ar ion sputtering for 10 and 20 s, indicating a decrease in amount of SiC from the shell to the core. The peaks at 99.8 and 103.3 eV shown in Figure 2c correspond to the binding energies of the Si2*p* electron in SiC and SiO_x_, respectively. The intensity of the peak at 99.8 eV decreases after the surface has been removed by Ar ion bombardment for 10 and 20 s, which is in agreement with the changes of C1*s* in SiC (Figure 2b). As a result, SiC and SiO_x_ exist mainly in the shell of the nanocapsules. The formation of SiC may be attributed to the plasma radiation reaction of SiO_2_ with C, following the reactions [7]:

(1) $$C_{2}H_{5}\;OH\to 2C+H_{2}O+2H_{2}\text{,}$$

(2) $$SiO_{2}+\left(2-x\right)\;C\to SiO_{x}+\left(2-x\right)\;CO\text{,}$$

(3) $$SiO_{x}+\left(1+x\right)C\to SiC+xCO$$

**Figure 3:**
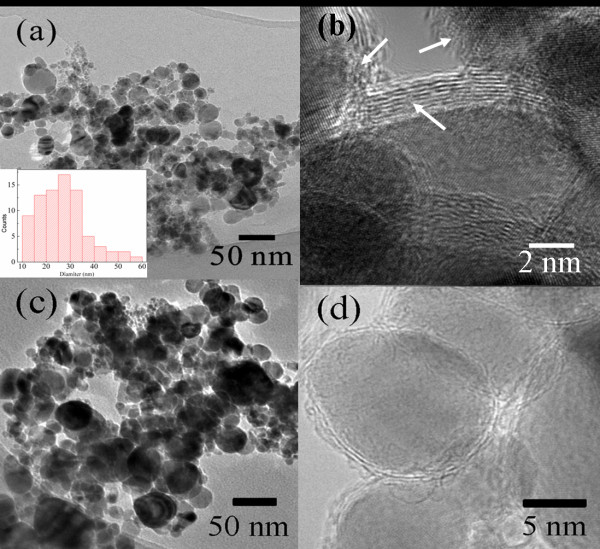
TEM and high-resolution TEM images of (a, b) Ni/(C, silicides) and (c, d) Ni/C nanocapsules.

The morphology and microstructure are presented in Figure 3a,b for Ni/(C, silicides) nanocapsules and in Figure 3c,d for Ni/C nanocapsules. The Ni/(C, silicides) nanocapsules with a core/shell microstructure are of a nearly spherical shape. Eighty nanoparticles have been measured on Ni/(C, silicides) nanocapsules, and the diameter distribution histogram is given as the inset of Figure 3a. The average diameter is 27 nm through statistical analysis. The distribution of the particle size and the shell thickness of Ni/(C, silicides) nanocapsules and Ni/C nanocapsules are similar, and the average thickness of the shells is 2 to 3 nm. It is noted that this value is much smaller than that (5 to 6 nm) for Ni/C nanocapsules [3]. The shell of Ni/C nanocapsules (Figure 3d) has a good layered structure with a distance between the layers of about 0.34 nm, which agrees with the interplanar spacing of the (002) plane of graphite. Compared with Ni/C nanocapsules, the shells of the Ni/(C, silicides) nanocapsules show a poor graphite layered structure and contain a large amount of amorphous phases indicated by the arrows in Figure 3b, which should be some amorphous carbon and silicides in the forms of SiO_x_ and SiC revealed by the XPS analysis above.

**Figure 4:**
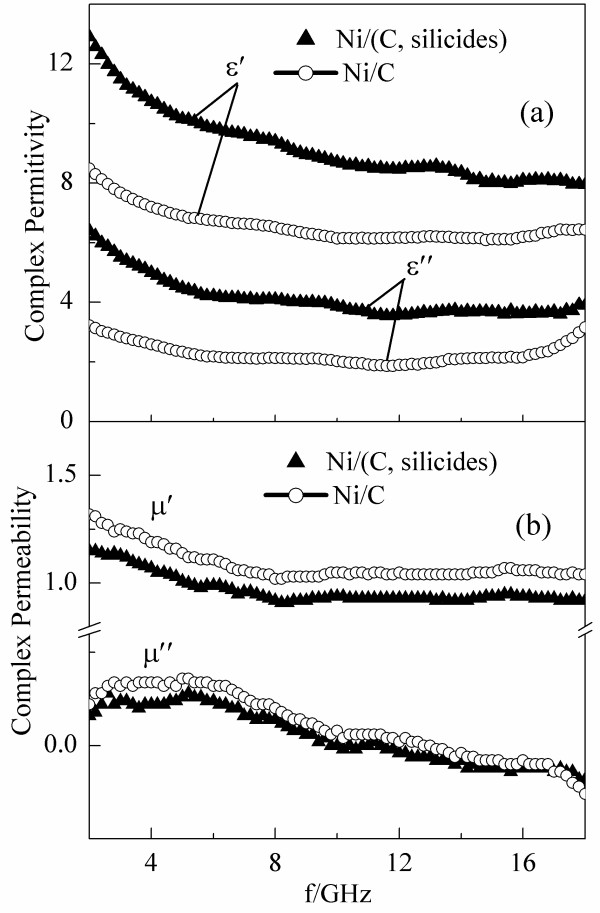
(a) Complex permittivity and (b) complex permeability of Ni/(C, silicides) nanocapsule-paraffin and Ni/C nanocapsule-paraffin composites.

Figure 4 shows the frequency dependence of the complex permittivity (*ε*’ + i*ε*”) and permeability (*μ*’ + i*μ*”) of Ni/(C, silicides) and Ni/C nanocapsules dispersed in paraffin in the frequency range between 2 and 18 GHz. According to Figure 4a, both real and imaginary parts of the complex permittivity of Ni/(C, silicides) and Ni/C nanocapsule-paraffin composites exhibit a similar tendency, i.e., declining significantly from 2 to 6 GHz and then decreasing slightly at higher frequencies. However, compared with those of the Ni/C nanocapsule-paraffin composite, both real and imaginary parts of the complex permittivity of the Ni/(C, silicides) nanocapsule-paraffin composite have larger values due to the doping with silicides, because SiC itself is a kind of dielectric material with a permittivity as high as 9.7 [12]. The inclusion of silicides and defects, such as lattice imperfections, in the shells of Ni/(C, silicides) nanocapsules indicated by the XPS analysis and HRTEM observation would create additional states near the Fermi level [13], and the defects themselves can be the polarization centers which localize a large amount of bound electrons [14,15]. The frequency dependences of complex permeability of the Ni/(C, silicides) and Ni/C nanocapsule-paraffin composites are shown in Figure 4b. The real part of the complex permeability (*μ*’) of the Ni/(C, silicides) nanocapsule-paraffin composite decreases from 1.15 to 0.9 and then remains almost constant in the frequency range of 8 to 18 GHz, while the imaginary part (*μ*”) increases slightly at 2 to 5 GHz and then decreases gradually at high frequencies. The Ni/C nanocapsule-paraffin composite has a similar tendency, but the value is larger by 0.1 on average for both real and imaginary parts. The decrease of the permeability of the Ni/(C, silicides) nanocapsule-paraffin composite can be explained by the introduction of inclusions of nonmagnetic silicides in the Ni/(C, silicides) nanocapsules.

**Figure 5:**
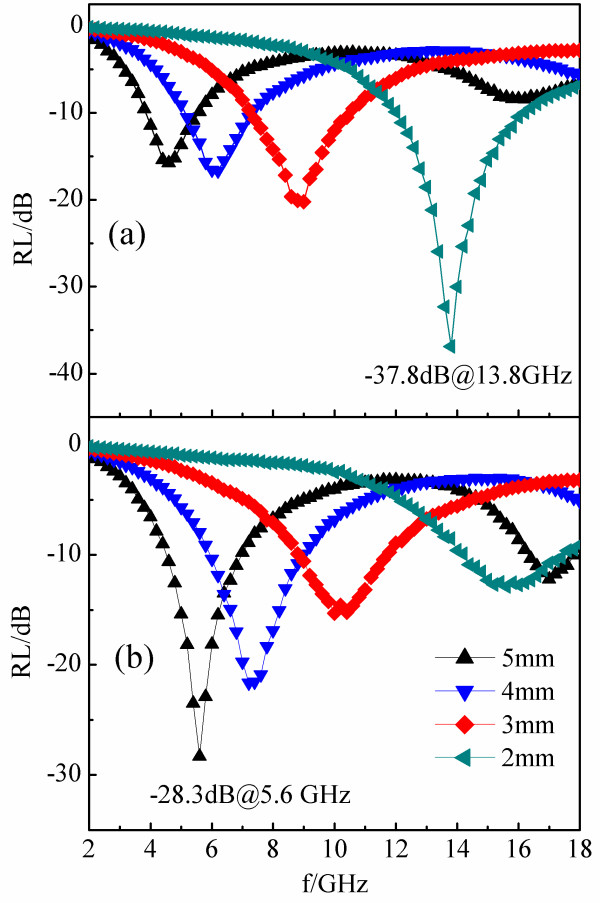
Calculated RL curves for (a) Ni/(C, silicides) nanocapsule-paraffin and (b) Ni/C nanocapsule-paraffin composites.

**Figure 6:**
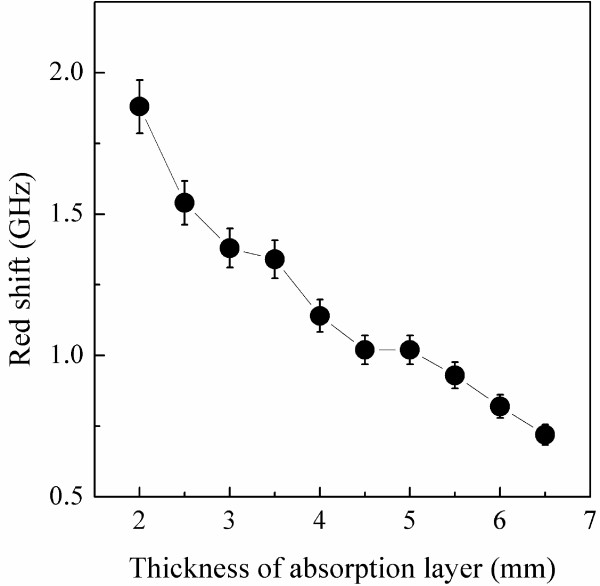
The difference of maximum RL peak positions between Ni/(C, silicides) nanocapsule-paraffin and Ni/C nanocapsule-paraffin composites.

Figure 5 shows the RL curves of Ni/(C, silicides) and Ni/C nanocapsules-paraffin composites. The RL values were calculated by means of the following equations [11]:

(4) $$\begin{array}{l} Z_{\mathrm{in}}=Z_{0}\;{\left(\frac{\mu_{r}}{\varepsilon_{r}}\right)}^{1/2}\tanh\left[j\left(\frac{2\pi fd}{c}\right)\;{\left(\mu_{r}\varepsilon_{r}\right)}^{1/2}\right]\text{,}\;\\ RL=20\log\left|\frac{Z_{\mathrm{in}}-Z_{0}}{Z_{\mathrm{in}}+Z_{0}}\right|\text{,} \end{array}$$

where *f* is the microwave frequency, *d* is the thickness of the sample, *c* is the velocity of light, *Z*_0_ is the impedance of free space, and *Z*_in_ is the input impedance. Figure 5a shows that the maximum absorption value of Ni/(C, silicides) nanocapsules with a thickness of 2 mm is −37.8 dB at 13.8 GHz, while the value of Ni/C nanocapsules is −12.9 dB at 15.6 GHz for the same thickness (Figure 5b). Compared with Ni/C nanocapsules, the absorption peaks of the Ni/(C, silicides) nanocapsules at the same thickness have shifted to a lower frequency, i.e., red shift. Figure 6 presents the difference of maximum RL peak positions between the Ni/(C, silicides) and the Ni/C nanocapsules at the different thicknesses of the absorption layer, in which the red shift value drops from 1.9 to 0.7 GHz as the thickness increases from 2 to 6.5 mm.

The distance between adjacent nanoparticles may play an important role in the electromagnetic response of Ni nanocapsule-paraffin composites [4]. According to [3], the inter-particle distance can be estimated by $s\;\text{=}\;D\left[{\left(\frac{\sqrt{2}\pi }{6\varphi }\right)}^{1/3}-1\right]$, where *D* is the average diameter of the nanoparticles and φ is the space occupancy ratio of the nanocapsules in paraffin. If the average particle size *D* is taken as 27 nm and φ = 50 wt.% for Ni/(C, silicides) nanocapsules, the inter-particle distance is about 25 nm for the Ni/(C, silicides) system. This inter-particle distance is much larger than the critical distance of about 11 nm [4]. As a result, the interaction between the Ni cores in Ni/(C, silicides) nanocapsules can be ignored, and only individual cores are involved during the microwave absorption process. This assumption is critical for the following discussion because band theory requires the negligence of inter-particle interaction. Belavin et al. [13] have calculated the electronic structure of defect and defect-free CNTs using the tight-binding method, and their results show that lattice defects can create localized states near the Fermi level. For Ni/(C, silicides) nanocapsules, the inclusion of silicides introduces large amounts of defects into the shells, such as amorphous silicides and carbon, vacancies, and twists in the layered carbon network. These defects in the shells of Ni/(C, silicides) nanocapsules cause the localization of electron density and generate additional energy levels near the Fermi level, which may reduce the electron transition energy. When the electromagnetic wave penetrates into absorbents, the wave is absorbed through electron transitions from quasi-continuous states induced by the defects near the Fermi level [14]. Because of the reduction of the electron transition energy, the transition can occur at a lower frequency, which leads to the red shift of the absorption peak at different thicknesses of absorption layer.

**Figure 7:**
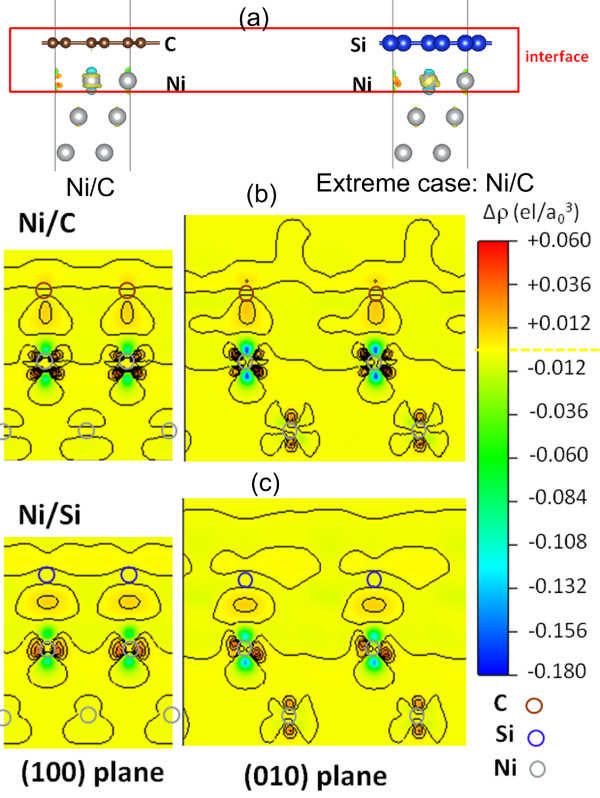
**(a) Schematic structures of two extreme cases for Ni-C and Ni-Si interfaces.** Charge transfer for (**b**) Ni-C and (**c**) Ni-Si electric polarizations. The color bar from blue via yellow to red refers to areas that are positively charged, neutrally charged, and negatively charged, respectively.

Here, we propose another possible mechanism, named the ‘electric dipole model’, to explain the red shift of the absorption peaks from Ni/C nanocapsules to Ni/(C, silicides) nanocapsules at the same thickness of absorption layer. We use an interface between two infinite slabs to simulate the interface between the Ni core and the graphite shell. Schematic structures for two extreme cases in this model are shown in Figure 7a. One is an infinite carbon layer that covers Ni (111), and the other is that a silicon layer is used instead of the carbon layer. In this simple model, a similar picture of charging density is observed in areas far away from the interface for both cases, charge transfer at the interface gives rise to an electric dipole (Figure 7b,c), and the electric dipole will vibrate upon microwave resonance absorption. This model, based on the electronegativity difference between C and Si, predicts a larger charge transfer between Ni and C than that between Ni and Si. When Si is introduced into the carbon shell of Ni/C nanocapsules, substituting Ni-C bonds for Ni-Si bonds, a smaller charge transfer occurs, and therefore, a reduced electric polarization in the Ni/Si case is found which results in the red shift of absorption peaks. The electric polarization or dipole moment is given by the equation:

(5) $$P=\int \left(r-R_{\mathrm{center}}\right)\rho \;d^{3}r\text{,}$$

where *ρ* is the electron density. The calculated dipole moment is 0.33 Debye for Ni-C and 0.31 Debye for Ni-Si (1 Debye = 3.336 × 10^−30^ C·m). An absorption frequency is estimated as follows:

(6) $$\omega =\sqrt{\frac{P\cdot E}{m\;d^{2}}\text{,}}$$

where *ω* is the vibration frequency of the electric dipole in response to the electric field; ***P***, the electric dipole; ***E***, the electric field strength; *m*, the effective mass; and *d*, the length of the electric dipole. A rough estimation shows that the order of magnitude of vibration (absorption) frequency *ω* falls in the gigahertz frequency range. Taken the calculated dipole moment for Ni-C and Ni-Si, $\Delta \omega =\frac{\omega_{Ni-C}-\omega_{Ni-Si}}{\omega_{Ni-C}}$ is estimated to be 26%. From the size distribution histogram of the Ni/(C, silicides) nanocapsules (the inset of Figure 3a), one can easily see that small particles (the particle size less than 40 nm) dominate in the Ni/(C, silicides) nanocapsules. As the thickness of the absorption layer of the Ni/(C, silicides) nanocapsule-paraffin composite increases, more small particles exist in the composite, which will decrease the red shift value because the absorption frequency *ω* is inversely proportional to the length of the electric dipole *d* according to Equation 2. It is why the difference of maximum absorption peaks between the Ni/(C, silicides) and Ni/C nanocapsule composites declines from 1.9 to 0.7 GHz as the thickness increases from 2 to 6.5 mm. It is suitable for our experimental result that the red shift varies from 10% to 20% at different layer thicknesses because of the mixture of electric polarization for Ni-C and Ni-Si. The XPS spectrum indicates that Si is distributed more in the outer carbon shell, and therefore, a more complicated structure model seems necessary to simulate the electric dipoles. However, a similar red shift phenomenon is anticipated because the dipole moment at the C-Si interface will compensate the dipole moment at the C-Ni interface and therefore will reduce the size of the dipole moment for Si-intercalated Ni/C nanocapsules.

## Conclusions

We have prepared Ni/(C, silicides) nanocapsules and Ni/C nanocapsules. XPS measurements reveal that the silicides mainly occur in the shells of nanocapsules. The two kinds of nanocapsule-paraffin composites demonstrate attractive microwave absorption properties. When inclusions of silicides are introduced into Ni/(C, silicides) nanocapsules, the absorption peaks of the Ni/(C, silicides) nanocapsule-paraffin composite have a red shift of 0.7 to 1.9 GHz compared with those of the Ni/C nanocapsule-paraffin composite. We propose a simple electric dipole model which semi-quantitatively explains the red shift phenomenon. The RL peak value for the Ni/(C, silicides)-paraffin composite with a layer thickness of 2 mm has increased to about −37 dB at 13.8 GHz compared with that of −13 dB at 15.6 GHz for the Ni/C nanocapsules-paraffin composite.

## Competing interests

The authors declare that they have no competing interests.

## Authors’ contributions

JJJ carried out the study, analyzed the data and wrote the manuscript. HW and DYG were involved in the sample preparation. HHG and TY elaborated the model. WST observed the TEM images. DL conceived of the study, and its design, drafted the manuscript and awarded as the principle investigator by NSFC to support this work. SM was involved in the conception of the study. WL was involved in the experimental design and awarded as PI by NBRP of China to support this work. ZDZ was involved in the conception of the study, the research guidance, discussion and paper modification. All authors read and approved the final manuscript.

## Authors’ information

Dr DL is a professor in the Institute of Metal Research, the Chinese Academy of Sciences. His main research interests include: synthesis of nanocrystals with novel properties; magnetic nanocapsules and nanostructures; magnetic, electrical transport and electromagnetic-wave absorption properties of functional nanomaterials.
